# Investigating novel biomarkers of immune activation and modulation in the context of sedentary behaviour: a multicentre prospective ischemic stroke cohort study

**DOI:** 10.1186/s12883-021-02343-0

**Published:** 2021-08-16

**Authors:** Katinka Nordheim Alme, Torunn Askim, Jörg Assmus, Tom Eirik Mollnes, Mala Naik, Halvor Næss, Ingvild Saltvedt, Per-Magne Ueland, Arve Ulvik, Anne-Brita Knapskog

**Affiliations:** 1 Institute of Clinical Medicine (K1), University of Bergen, Bergen, Norway; 2 Department of Internal Medicine, Haraldsplass Deaconess Hospital, Bergen, Norway; 3 Department of Neuromedicine and Movement Science, Faculty of Medicine and Health Science, NTNU-Norwegian University of Science and Technology, Trondheim, Norway; 4 Centre for Clinical Research, Haukeland University Hospital, Bergen, Norway; 5 Department of Immunology, Oslo University Hospital and University of Oslo, Oslo, Norway; 6 Research Laboratory, Nordland Hospital, Bodø, and K.G. Jebsen TREC, University of Tromsø, Tromsø, Norway; 7 Centre of Molecular Inflammation Research, Norwegian University of Science and Technology, Trondheim, Norway; 8 Department of Clinical Science (K2), University of Bergen, Bergen, Norway; 9 Department of Neurology, Haukeland University Hospital, Bergen, Norway; 10 Centre for age-related medicine, Stavanger University Hospital, Stavanger, Norway; 11 Department of Geriatrics, Clinic of internal medicine, St Olavs Hospital, Trondheim University Hospital, Trondheim, Norway; 12 Bevital AS, Bergen, Norway; 13 Department of Geriatric Medicine, Oslo University Hospital, Ullevaal, Oslo, Norway

**Keywords:** Sedentary behavior, Inflammation, Immune modulation, Vascular disease, Kynurenine pathway, Stroke

## Abstract

**Background:**

Sedentary behaviour is associated with disease, but the molecular mechanisms are not understood. Valid biomarkers with predictive and explanatory properties are required. Therefore, we have investigated traditional and novel biomarkers of inflammation and immune modulation and their association to objectively measured sedentary behaviour in an ischemic stroke population.

**Methods:**

Patients admitted to hospital with acute ischemic stroke were included in the multicentre Norwegian Cognitive Impairment After Stroke (Nor-COAST) study (*n* = 815). For this sub-study (*n* = 257), sedentary behaviour was registered 3 months after stroke using position transition data from the body-worn sensor, ActivPal®. Blood samples were analysed for high sensitive C-reactive protein (hsCRP), the cytokines interleukin-6 (IL-6) and 10 (IL-10), neopterin, tryptophan (Trp), kynurenine (kyn), kynurenic acid (KA), and three B6 vitamers, pyridoxal 5′-phosphate (PLP), pyridoxal (PL), and pyridoxic acid (PA). The kynurenine/tryptophan ratio (KTR) and the pyridoxic acid ratio index (PAr = PA: PL + PLP) were calculated.

**Results:**

Of the 815 patients included in the main study, 700 attended the three-month follow-up, and 257 fulfilled the inclusion criteria for this study. Sedentary time was significantly associated with levels of hsCRP, IL-6, neopterin, PAr-index, and KA adjusted for age, sex, waist circumference, and creatinine. In a fully adjusted model including all the significant biomarkers except hsCRP (because of missing values), sedentary time was independently positively associated with the PAr-index and negatively with KA. We did not find an association between sedentary behaviour, IL-10, and KTR.

**Conclusions:**

The PAr-index is known to capture several modes of inflammation and has previously shown predictive abilities for future stroke. This novel result indicates that the PAr-index could be a useful biomarker in future studies on sedentary behaviour and disease progression. KA is an important modulator of inflammation, and this finding opens new and exciting pathways to understand the hazards of sedentary behaviour.

**Trial registration:**

The study was registered at Clinicaltrials.gov (NCT02650531). First posted 08/01/2016.

**Supplementary Information:**

The online version contains supplementary material available at 10.1186/s12883-021-02343-0.

## Background

Sedentary behaviour is associated with an increased risk of vascular disease, amongst others, through inflammatory pathways [1–5]. The impact depends on the properties of the sedentary behaviour and the corresponding physical activity, which can vary in bout length, intensity, and habit [1, 6–9]. To increase the understanding of the link between sedentary behaviour and vascular disease, valid biomarkers with predictive and explanatory properties are required.

Cytokines can be predominantly pro- or anti-inflammatory, but their downstream effect also depends on other contextual factors. Also, cytokines vary in their biological and analytical properties, and some of them, such as interleukin 1β (IL-1β) and interferon ɣ (IFN-ɣ), are therefore often measured indirectly by other more stable downstream molecules [5, 10, 11]. The inflammatory biomarker C-reactive protein (CRP) is a downstream marker of interleukin 6 (IL-6) and IL-1β [10]. The inflammation associated with this pathway has been shown to increase with age and to be associated with sedentary behaviour and disease development [1, 2, 10, 12–14]. Interleukin-10 (IL-10) is a predominantly anti-inflammatory cytokine found to be induced by acute bouts of moderate to vigorous physical activity (MVPA) and in response to chronic exercise [4, 15–17]. The association to sedentary behaviour is not clear.

The inflammatory pathway associated with IFN-ɣ can be measured by the ratio between kynurenine and tryptophan—the kynurenine tryptophan ratio (KTR)—and neopterin secreted by activated macrophages. Kynurenine is a metabolite of the amino acid tryptophan and is the first metabolic step of the kynurenine pathway (KP) [18, 19]. KTR and neopterin have been shown to predict future coronary events [11, 20], but the association to sedentary behaviour is unclear [21, 22].

A novel and sensitive biomarker of inflammation, the pyridoxal acid ratio index (PAr-index = (pyridoxic acid (PA): (pyridoxal (PL) + pyridoxal 5-phosphate (PLP)), represents several modes of inflammation and captures the effect of the inflammatory pathways associated with CRP, white blood cell count, neopterin, and KTR [23, 24]. The PAr-index has been shown to predict future stroke with better precision than high sensitive C-reactive protein (hsCRP) [25] but has never been studied in the context of sedentary behaviour.

Kynurenic acid (KA), a side-product in the KP, is not an inflammatory biomarker but part of a negative feedback mechanism inducing immune tolerance [26, 27]. The role of KA in health and disease is not fully understood [26] and KA has never been investigated in relation to sedentary behaviour. KA has been found to increase following physical exercise and KA can potentially be one of the molecular links between sedentary behaviour and inflammation [22, 28–32].

The primary objective of the present study was to investigate the association between novel biomarkers of inflammation and immune modulation and objectively measured habitual sedentary behaviour in a stroke population. The secondary objective was to investigate the impact of bout duration on these associations. Our hypothesis was that sedentary behaviour would be positively associated with a pro-inflammatory profile.

## Material and methods

### Subjects

The prospective cohort study, the Norwegian Cognitive Impairment After Stroke Study (Nor-COAST), included 815 adults admitted for acute stroke at one of five contributing hospitals from May 2015 through March 2017. Details of the Nor-COAST study, including inclusion and exclusion criteria and patient selection, have been published elsewhere [33, 34]. In the current study, only patients with ischemic stroke who attended the three-month follow-up, had blood samples drawn for direct analyses at the local laboratory and for storage in the bio-bank, were able to walk 50 m independently or with support from a person/walker (Barthel index item 9, ≥10 points), and had valid activity data were included.

### Clinical data and laboratory analyses

Demographic information, as well as information about risk factors of vascular disease, stroke severity, and functional outcomes were collected during the index stay and at the three-month follow-up. Stroke severity was measured by the National Institute of Health Stroke Scale (NIHSS) at the time of hospital arrival and at three-month follow-up [35]. Functional state was measured by the Barthel index and the modified Rankin scale at discharge or day seven. The Barthel index is a 10-item assessment for basal activities of daily living, with a maximum score of 100 points indicating no functional deficits [36]. The modified Rankin scale is a five-point scale measuring global disability, where 0–2 is defined as ‘good outcomes’, 3–5 indicates increasingly disability, and 6 is death [37].

#### Laboratory markers

*Non-fasting blood samples were collected at the three-month follow-up at the outpatient clinic.*

##### Routine clinical-chemical analyses

The blood samples were analysed for high sensitive C-reactive protein (hsCRP, mg/L) and creatinine (μmol/L) at the local laboratory directly. The estimated glomerular filtration rate (eGFR) was calculated with the Chronic Kidney Disease Epidemiology Collaboration formula [38] using a dedicated STATA software package.

##### Sample collection of research analyses

Aliquots of serum and plasma were immediately frozen at − 80 °C at the inclusion hospital. The samples were transported on dry ice and stored at BioBank1, Central Norway Health Authority. In 2019, two aliquots of plasma were transported on dry ice to the Research Laboratory Nordland Hospital (Bodø, Norway) and Bevital A/S (Bergen, Norway), respectively, and were thawed only once.

##### Cytokines

Interleukin (IL)-6 and IL-10 were measured as the classical cytokines previously shown to be of interest in these patients (see Introduction). They were analysed at the Research Laboratory Nordland Hospital using the Bio-Plex technology kits obtained from Bio-Rad Laboratories Inc., Hercules, CA. The assay was performed according to the manufacturer’s procedure. Values below the lower detecting limit (*n* = 36 for IL-10, none for IL-6) were replaced by random values below the lower detecting limit when statistics were performed.

##### Other biomarkers

The three vitamin B6 forms pyridoxal 5′-phosphate (PLP), pyridoxal (PL), and 4-pyridoxic acid (PA) were measured along with tryptophan, the two tryptophan metabolites kynurenine and kynurenic acid (KA), and neopterin as novel biomarkers of interest (see Introduction) as part of analytic platform D at Bevital A/S (Bergen, Norway) by liquid chromatography/tandem mass spectrometry using EDTA-plasma. The PAr-index was calculated as PA:(PL + PLP) [23].

### Sedentary behaviour

Sedentary behaviour was defined as sitting or lying. Time in sedentary behaviour was given as total time per day and as time accumulated through pre-defined bout-length categories (< 30 min, 30–59 min, 60–89 min, and ≥ 90 min). Sedentary behaviour was measured by registering position transition with a single thigh-worn sensor (ActivPal3®, Model 20.2, PAL Technologies Ltd., Glasgow, United Kingdom); in patients with hemiparesis, the sensor was attached to the unaffected side. The monitor was attached when the patient visited the outpatient clinic and returned by mail.The threshold for noise was 1.5 s, and sedentary events were merged if they were interrupted by events lasting ≤3 s. Measurements were performed for seven consecutive days/nights and analysed using a custom-made MATLAB® script (version R2016b, MathWorks, Natick, MA, USA) to extract periods of sedentary behaviour from the recordings. The recordings were analysed for daytime sedentary behaviour, defined as occurring from 08:00 am through 10:00 pm, and a registered period of sedentary behaviour—a sedentary bout—was divided into two periods if it crossed these time boundaries. Only patients with recordings from at least four full 24-h periods starting from 08:00 am were included. As a final control, manual inspection of the output to identify non-wear time was performed.

### Statistics

Laboratory results and characteristics at baseline and three months were given in means and standard deviations, numbers with percentages, and medians with interquartile range. Differences between groups were tested with the chi-squared test, t-test, and Wilcoxon-Mann-Whitney test.

Sedentary behaviour was given as a continuous variable of mean hours per day, both as total time of sedentary behaviour and as time accumulated through different bout-length categories (< 30 min, 30–59 min, 60–89 min, and ≥ 90 min).

Associations between variables were assessed using multiple linear and Tobit regressions using the biomarker as the dependent variable and sedentary behaviour as the independent variable adjusted for age, sex, waist circumference, and kidney function [27, 39–41]. The results were given as standardised beta coefficients with confidence intervals and *p*-values. One final regression was made to investigate the independent association between the biomarkers significant in the initial analyses and sedentary behaviour. Here sedentary behaviour was used as the dependent variable, while age, sex, waist circumference, and kidney function were independent variables, in addition to the biomarkers with significant results in the primary analyses, with the exception of hsCRP because of missing values (see below). A sensitivity analysis including only those patients with values for hsCRP was added. The residuals of the regressions did not follow a normal distribution, and the value of the biomarkers were log-transformed. For the cytokines, the significance test was performed using a Tobit regression with left censoring [41], applying the highest value amongst the lower detection limits as cut-off (see laboratory analyses for details). The analyses were performed in STATA/SE 16.1.

In the final population, 88 cases had missing values for hsCRP because this analysis was not available at two of the inclusion centres; 12 had missing values for waist circumference and 4 for eGFR.

## Results

### Patient characteristics and laboratory values

**Fig. 1:**
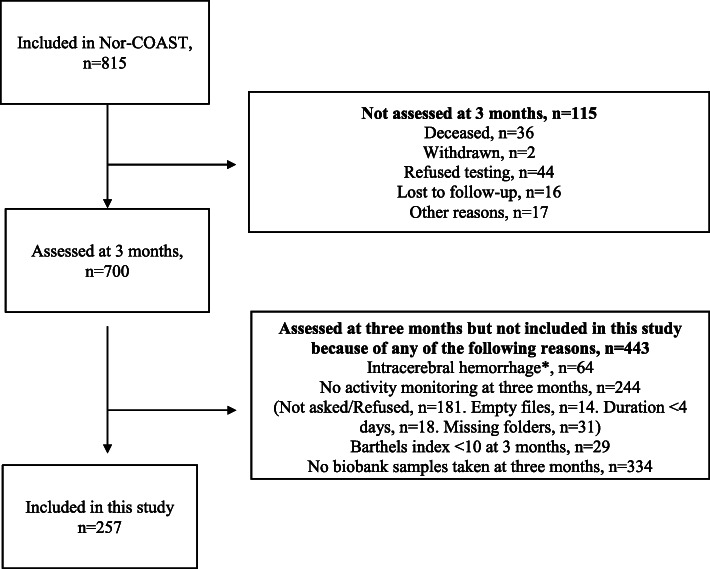
Patient selection

Out of 700 patients attending three-month follow-up, 257 fulfilled the inclusion criteria (Fig. 1). Of the included patients, 149 (58%) were men; mean age was 73 (±11) years; they had a mean body mass index (BMI) of 27 (±4.3) and a waist circumference of 95 (±13). Compared to the rest of the Nor-COAST population, our subgroup had a lower NIHSS on admission (3.7 (4.6) vs. 5.0 (6.5), *p* = 0.004) and at 3 months (0.7 (1.3) vs. 1.0 (2.0), *p* = 0.022), had more favourable functional outcomes (Barthel index 98 (6.1) vs. 90 (21), *p* < 0.001) and scores for global disability (modified Rankin scale (1.5 (0.9) vs. 2.0 (1.4), p < 0.001) at 3 months. There were no differences in the laboratory results between the patient groups, except for the PAr-index, which was higher in included patients (0.64 (0.44, 0.91) vs. 0.52 (0.39, 0.73), *p* = 0.005) (Table 1).

**Table 1:** Baseline and three-month characteristics of the study population compared to the remaining Nor-COAST population

	Total *n* = 700		
	Not included (*n* = 443)	Included (*n* = 257)	p
Sex, male, n (%)	251 (57)	149 (58)	0.734
Age at three months, mean y (SD)	74 (12)	73 (11)	0.162
BMI at three months, mean kg/m^2^ (SD)	27 (4.2)	27 (4.3)	0.797
Waist at three months, mean cm (SD)	97 (13)	95 (13)	0.283
NIHSS on admission, mean (SD)	5.0 (6.5)	3.7 (4.6)	0.004
At three months	1.0 (2.0)	0.7 (1.3)	0.022
Modified Rankin scale, mean (SD)	2.5 (1.5)	2.0 (1.1)	< 0.001
At three months	2.0 (1.4)	1.5 (0.9)	< 0.001
Modified Rankin scale ≤2, n (%)	269 (49)	178 (70)	< 0.001
At three months	304 (68)	224 (88)	< 0.001
Barthel index, mean (SD)	79 (28)	92 (14)	< 0.001
At three months	90 (21)	98 (6.1)	< 0.001
Laboratory values at three months*, median (IQR)			
eGFR (*N* = 272/253)	77 (60, 89)	76 (63, 88)	0.696
hsCRP (*N* = 98/169)	1.9 (0.7, 3.6)	1.6 (0.8, 3.7)	0.513
IL6 (*N* = 111/257)	4.6 (2.5, 6.7)	4.6 (2.9, 7.4)	0.438
IL10 (*N* = 111/257)	18 (8.6, 32)	20 (8.6, 36)	0.304
Neopterin (*N* = 109/257)	16 (12, 22)	16 (12, 22)	0.998
PAr-index (*N* = 109/257)	0.52 (0.39, 0.73)	0.64 (0.44, 0.91)	0.005
KTR (*N* = 109/257)	32 (28, 40)	34 (29, 42)	0.062
KA (*N* = 109/257)	55 (44, 75)	59 (46, 76)	0.276

^*^Number of patients attending baseline and three months: A total of 558 patients had blood samples taken at three months. Of these, 368 had biobank samples taken. The N for each individual biomarker is shown for each group (not included/included)

### Sedentary behaviour

**Fig. 2:**
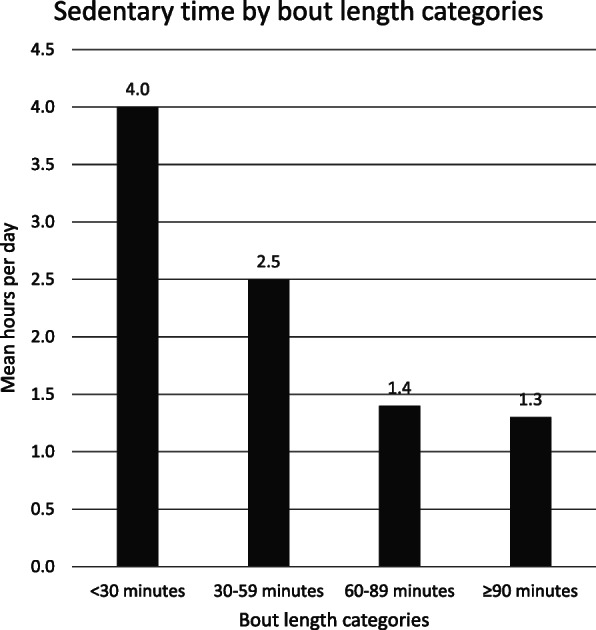
Mean distribution of daytime sedentary time (from 08:00 am to 10:00 pm)

The patients in our study had a mean total sedentary time of 9.7 (1.9) hours per day. Of the total time spent sedentary, 4.0 (1.1) hours were accumulated through bouts of less than 30 min, 2.5 (0.9) hours through bouts of 30–59 min, 1.4 (0.9) hours through bouts of 60–89 min, and 1.3 (1.5) hours through bouts ≥90 min (Fig. 2).

### Regression analyses

In the adjusted analyses we found hsCRP (0.25, *p* = 0.001), IL-6 (0.17, *p* = 0.009), neopterin (0.12, *p* = 0.034), and the PAr-index (0.21, *p* < 0.001) to be positively associated with total sedentary time (Table 2). The KA (− 0.10, *p* = 0.045) showed an inverse association with sedentary time. No significant associations were found for IL-10 and KTR.

**Table 2:** The association between biomarkers and total sedentary time

	Crude			Adjusted^a^		
	β	95%CI	P	β	95%CI	P
hsCRP						R^2^ = 0.16
Total sedentary time	0.32	(0.18, 0.47)	< 0.001	0.25	(0.09, 0.39)	0.001
IL-6^b^						R^2^ = 0.12
Total sedentary time	0.26	(0.14, 0.38)	< 0.001	0.17	(0.04, 0.30)	0.009
IL-10^b^						R^2^ = 0.04
Total sedentary time	0.04	(−0.09, 0.16)	0.509	0.01	(− 0.12, 0.14)	0.841
Neopterin						R^2^ = 0.35
Total sedentary time	0.24	(0.12, 0.36)	< 0.001	0.12	(0.00, 0.22)	0.034
PAr-index						R^2^ = 0.37
Total sedentary time	0.33	(0.22, 0.45)	< 0.001	0.21	(0.09, 0.30)	< 0.001
KTR						R^2^ = 0.45
Total sedentary time	0.21	(0.09, 0.33)	0.001	0.06	(−0.05, 0.15)	0.238
KA						R^2^ = 0.48
Total sedentary time	0.04	(−0.08, 0.16)	0.512	−0.10	(−0.21, − 0.02)	0.045

hsCRP = high sensitive C-reactive protein. IL-6 = interleukin-6. IL-10 = interleukin-10, PAr-index = (4-pyridoxic acid/(pyridoxal 5′-phosphate + pyridoxal). KTR = kynurenine/tryptophan ratio = (kynurenine nM/tryptophan μM), KA = kynurenic acid

^a^Model: biomarker is the dependent variable. Sedentary time, age, sex, waist circumference, and creatinine are independent variables

^b^For IL-6 and IL-10, Tobit regressions were used for the significance test and regular linear regressions to calculate the beta coefficients

In the final model, where all biomarkers significantly associated with sedentary time were added, only the PAr-index (0.25, *p* = 0.001) and KA (− 0.19, *p* = 0.021) remained independently associated with total sedentary time (Table 3). In a sensitivity analyses (Table 4) restricted to those with data on hsCRP, only the PAr-index (0.31, p = 0.001) were significantly associated. Adding hsCRP to this model did not change the results (numbers not shown).

**Table 3:** Adjusted multiple regression model of the association between total sedentary time and biomarkers, *n* = 242

Sedentary time	β	95% CI	P
IL-6	0.12	(−0.01, 0.24)	0.069
Neopterin	0.08	(−0.07, 0.23)	0.280
PAr-index	0.25	(0.10, 0.39)	0.001
Kynurenic acid	−0.19	(−0.35, − 0.03)	0.021
Age	0.16	(0.03, 0.29)	0.017
Sex (male)	0.01	(−0.28, 0.30)	0.941
Creatinine	0.02	(−0.17, 0.21)	0.826
Waist circumference	0.21	(0.08, 0.33)	0.002

IL-6 = interleukin-6. PAr-index = (4-pyridoxic acid/(pyridoxal 5′-phosphate + pyridoxal)

**Table 4:** Adjusted multiple regression model of the association between total sedentary time and biomarkers restricted to those with a value for hsCRP, *n* = 161

Sedentary time	β	95% CI	P
IL-6	0.10	(−0.06, 0.26)	0.229
Neopterin	0.09	(−0.11, 0.29)	0.374
PAr-index	0.31	(0.13, 0.49)	0.001
Kynurenic acid	−0.17	(−0.38, 0.04)	0.110
Age	0.11	(−0.05, 0.27)	0.195
Sex (male)	0.05	(−0.31, 0.40)	0.803
Creatinine	−0.05	(−0.30, 0.20)	0.692
Waist circumference	0.15	(−0.01, − 0.31)	0.062

IL-6 = interleukin-6. PAr-index = (4-pyridoxic acid/(pyridoxal 5′-phosphate + pyridoxal)

There was a tendency towards an increased association with longer sedentary bout lengths for hsCRP, IL-6, neopterin, and PAr-index (Supplementary Table 1).

## Discussion

In this study, we have explored novel biomarkers associated with inflammation and immune modulation and their association to objectively measured sedentary time in an ischemic stroke population 3 months after the acute stroke. We have also replicated known associations between traditional biomarkers of inflammation and sedentary behaviour. We found hsCRP, IL-6, neopterin, PAr-index and KA (invers) to be significantly associated with sedentary time. The PAr-index and KA also showed an independent association in a model including IL-6 and neopterin.

The PAr-index has never been studied in the context of sedentary behaviour. The PAr-index reflects several modes of inflammation and has been shown to be associated with CRP, white blood cell count, and markers of cellular immune activation such as neopterin and KTR [23]. In accordance to this, the independent association in the fully adjusted model illustrates that the PAr-index captures the effect of the other pathways by attenuating the association to IL-6 and neopterin when included in the same model. As the PAr-index has recently found to be associated with the risk of future stroke, it might be a valid and clinically relevant biomarker for future intervention studies on sedentary behaviour [25].

The association between sedentary time, hsCRP and IL-6 confirms prior findings, both from observational and intervention studies [1, 2, 8, 40, 42]. The results from intervention studies imply a causal relationship between sedentary behaviour and inflammation, and Noz et al. found a phenotypic shift in the innate immune system towards a less inflammatory phenotype of circulating monocytes in vitro when sedentary time was replaced with physical activity [1]. Other studies have found a change in the inflammatory phenotype of infiltrating inflammatory cells in adipose tissue in addition to a reduction in total adipose tissue volume in response to exercise [4, 5, 8, 40].

Neopterin and KTR are indirect measures of IFN-ɣ activity, which plays a central role in activating cellular immune response [19, 43]. Neopterin and KTR have been found to be associated with future major vascular events and all-cause mortality [11]. We found a significant association between sedentary behaviour and neopterin, but not KTR. A less convincing association between sedentary behaviour and biomarkers associated with IFN-ɣ-mediated inflammation might be explained by findings from the above-mentioned intervention study by Noz et al., where increased walking time altered innate immune function towards a less pro-inflammatory state in combination with an increased IFN-ɣ production capacity upon stimulation [1]. The implication of this in the context of inflammation and vascular disease, is not clear. This is the first study to investigate sedentary behaviour and its association with neopterin and KTR using objective measures for monitoring daytime activity. Prior investigations were predominantly based on questionnaire information and investigated the association to physical activity rather than to sedentary behaviour. The results were inconclusive [21, 22, 31].

We found an inverse association between KA and sedentary behaviour, and the association was strengthened after adjustment for inflammation. KA is metabolised from kynurenine by kynurenine amino transferases (KATs) [27] and is part of a negative feedback loop where chronic inflammation induces immune tolerance via the aryl hydrocarbon receptor (AhR) and the G-protein-coupled receptor 35 [18, 26, 27, 44, 45]. In a recent review, Joisten et al. argued for the relevance of KA in disease prevention [26]. KAT expression and activity has been found to be induced by chronic exercise via peroxisome proliferator-activated receptor-gamma coactivator 1 alpha (PGC1α) and the associated transcription factor, peroxisome proliferator activated receptor alpha (PPARα) [28–31]. The inverse relationship found in our study suggests a reduced KAT expression during sedentary behaviour. PPARα has been found to be negatively associated with IL-6 in older adults, and Lustgarten et al. observed that PPARα activation was related to decreased inflammation [12]. Kynurenic acid could be a molecular link between sedentary behaviour, inflammation, and vascular diseases such as stroke. The KA association was significant only after adjustment for kidney function. KA is eliminated in urine, and lower muscle mass is associated with lower creatinine and higher levels of sedentary behaviour [46]. This can explain the observed change after the adjustments. In the sensitivity analyses restricted to those with a value for hsCRP, the association to KA was no longer significant, probably due to lower sample size. The strengthened association between sedentary behaviour and KA, independent of inflammation in the final model, can be seen as a ‘proof of concept’ of the connection between sedentary behaviour and immune modulation.

Prior studies have found increased levels of the anti-inflammatory cytokine IL-10 in response to exercise [4, 15–17]. A single bout of MVPA has been associated with an increased expression of IL-6 from the myocytes, and this spike of IL-6 is believed to induce IL-10 [4]. We expected an inverse association between sedentary behaviour and IL-10. The lack of association between sedentary time and IL-10 in our study might be explained by the distinction between physical activity in general (the inverse of sedentary behaviour) and MVPA, which has a higher level of energy expenditure. This also illustrates how duration and intensity of cytokine expression is important for the downstream effects. IL-6 falls to basal levels within an hour after exercise [4], and this dual role of IL-6 prompts caution when designing intervention studies.

From our results, it seems that all sedentary behaviour was associated with increased inflammation, but that there was a trend towards stronger associations to sedentary time accumulated through longer bouts. In some cases, the association between sedentary behaviour and the biomarker was no longer significant when stratified for bout length because of reduced statistical power, which also potentially explain the spread in the results.

The strength of this study lies in the sample size, the carefully characterised population of older patients with vascular disease, and the objective measure of sedentary behaviour over several days in a habitual setting. The Nor-COAST population is representative of the group of patients who have suffered mild strokes [34]. The use of both traditional and novel biomarkers has enabled us to confirm prior findings, contributing to filling in the gaps of uncertainties and to opening new and interesting paths and new perspectives. Information about other explanatory variables such as age, waist circumference, and kidney function have increased the validity of the results.

Because of the observational nature of the study, we can only identify associations and not any causal direction. Methods of measuring, defining, and analysing sedentary behaviour were thoroughly discussed in a prior study [47], however, because sedentary bouts were cut if they crossed the day/night timeline, some overrepresentation of shorter bout lengths and concordant underestimation/to the expense of longer bout lengths may have occurred. As shown in the baseline table, the stroke patients in this study were fitter compared to the entire Nor-COAST population, and the findings cannot be generalised to the entire stroke population.

## Conclusion

This novel result indicates that the PAr-index is a potentially useful biomarker in future studies on sedentary behaviour and disease progression. The association to KA opens fresh interesting pathways to understanding disease progression in general, the hazards of sedentary behaviour in particular, and should be further investigated.

## Supplementary Information

**Additional file 1: Supplementary Table 1**. Crude and adjusted linear regression analyses of the association between biomarkers and time in sedentary behaviour by bout-length category.

## Data Availability

Due to Norwegian regulations and conditions for informed consent, the dataset is not publicly available.

## Abbreviations

AhR: Aryl hydrocarbon receptor
BMI: Body mass index
CRP: C-reactive protein
eGFR: Estimated glomerular filtration rate
hsCRP: High sensitive C-reactive protein
IFN-ɣ: interferon gamma
IL-6: Interleukin-6
IL-10: Interleukin-10
KA: Kynurenic acid
KAT: Kynurenine aminotransferases
KP: Kynurenine pathway
KTR: Kynurenine/tryptophan ratio
MVPA: Moderate to vigorous physical activity
NIHSS: National Institute of Stroke Scale
Nor-COAST: Norwegian Cognitive Impairment After Stroke Study
PAr-index: Pyridoxal acid ratio index
PLP: Pyridoxal-5-phosphate
PL: Pyridoxal
PA: Pyridoxal acid
PGC1α: Peroxisome proliferator-activated receptor-gamma coactivator 1 alpha
PPARα: Peroxisome proliferator-activated receptor alpha
REK: Regional ethics committee
