# Supplementary material for: Investigating novel biomarkers of immune activation and modulation in the context of sedentary behaviour: a multicentre prospective ischemic stroke cohort study
Source: BMC Neurol. 2021 Aug 16;21:318. doi: 10.1186/s12883-021-02343-0 (PMC8365944; doi:10.1186/s12883-021-02343-0)
Supplement: Supplementary file 1 — Additional file 1: Supplementary Table 1. Crude and adjusted linear regression analyses of the association between biomarkers and time in sedentary behaviour by bout-length category. [file 12883_2021_2343_MOESM1_ESM.pdf]

Supplementary file

Title page

Investigating novel biomarkers of immune activation and modulation in the context of sedentary behaviour: a multicentre prospective ischemic stroke cohort study.

Authors

Alme, Katinka Nordheim[1, 2.]

Askim, Torunn[3.]

Assmus, Jörg[4]

Mollnes, Tom Eirik[5, 6, 7]

Naik, Mala [2, 8.]

Næss, Halvor [1, 9, 10.]

Saltvedt, Ingvild [3, 11.]

Ueland, Per-Magne[12]

Ulvik, Arve[12]

Knapskog, Anne-Brita[13.]

1) Institute of Clinical Medicine (K1), University of Bergen, Norway.
2) Department of Internal Medicine, Haraldsplass Deaconess Hospital, Bergen, Norway
3) Department of Neuromedicine and Movement Science, Faculty of Medicine and Health Science, NTNU-Norwegian University of Science and Technology, Trondheim, Norway.
4) Centre for Clinical Research, Haukeland University Hospital, Bergen, Norway.

5) Department of Immunology, Oslo University Hospital and University of Oslo, Oslo, Norway.
6) Research Laboratory, Nordland Hospital, Bodø, and K.G. Jebsen TREC, University of Tromsø, Norway.
7) Centre of Molecular Inflammation Research, Norwegian University of Science and Technology, Trondheim, Norway
8) Department of Clinical Science (K2), University of Bergen, Norway.
9) Department of Neurology, Haukeland University Hospital, Bergen, Norway
10) Centre for age-related medicine, Stavanger University Hospital, Stavanger, Norway
11) Department of Geriatrics, Clinic of internal medicine, St Olavs hospital, Trondheim University hospital, Trondheim, Norway
12) Bevital AS, Bergen, Norway
13) Department of Geriatric Medicine, Oslo University Hospital, Ullevaal, Oslo, Norway,

Corresponding author: Katinka Nordheim Alme

Email: katinka.alme@gmail.com

Twitter: @katinkaalme

Supplementary table 1: Crude and adjusted linear regression analyses of the association between biomarkers and time in sedentary behaviour by bout-length category.

	Crude			Adjusted		
	β	95% CI	P	β	95% CI	P
hsCRP			R^2=0.12			R^2=0.19
<30 minutes	0.15	(-0.02, 0.33)	0.092	0.12	(-0.05, 0.30)	0.171
30-59 minutes	0.21	(0.06, 0.36)	0.006	0.11	(-0.04, 0.27)	0.143
60-89 minutes	0.00	(-0.16, 0.17)	0.979	-0.06	(0.06, -0.18)	0.493
>90 minutes	0.32	(0.15, 0.49)	<0.001	0.34	(0.17, 0.51)	<0.001
IL6			R^2=0.07			R^2=0.13

<30 minutes	0.17	(0.01, 0.32)	0.032	0.15	(-0.01, 0.30)	0.053
30-59 minutes	0.12	(0.00, 0.25)	0.044	0.07	(-0.06, 0.20)	0.231
60-89 minutes	0.06	(-0.07, 0.20)	0.338	0.00	(-0.14, 0.14)	0.985
>90 minutes	0.27	(0.12, 0.41)	0.001	0.22	(0.07, 0.37)	0.005
IL10			R^2=0.01			R^2=0.05
<30 minutes	0.11	(-0.05, 0.27)	0.204	0.13	(-0.03, 0.29)	0.114
30-59 minutes	-0.02	(-0.15, 0.11)	0.876	-0.04	(-0.18, 0.09)	0.837
60-89 minutes	0.04	(-0.11, 0.18)	0.947	0.03	(-0.11, 0.18)	0.888
>90 minutes	0.06	(-0.09, 0.21)	0.331	0.04	(-0.13, 0.19)	0.535
Neopterin			R^2=0.07			R^2=0.38
<30 minutes	0.22	(0.06, 0.37)	0.006	0.20	(0.07, 0.33)	0.002
30-59 minutes	0.16	(0.04, 0.29)	0.008	0.07	(-0.07, 0.22)	0.178
60-89 minutes	0.08	(-0.06, 0.21)	0.285	-0.03	(-0.15, 0.09)	0.610
>90 minutes	0.20	(0.05, 0.34)	0.008	0.16	(0.04, 0.29)	0.012
PAr-index			R^2=0.12			R^2=0.38
<30 minutes	0.21	(0.06, 0.36)	0.007	0.20	(0.07, 0.33)	0.003
30-59 minutes	0.16	(0.04, 0.28)	0.008	0.07	(-0.04, 0.18)	0.201
60-89 minutes	0.27	(0.13, 0.40)	<0.001	0.17	(0.05, 0.29)	0.005
>90 minutes	0.18	(0.04, 0.32)	0.013	0.14	(0.01, 0.26)	0.032
Kynurenine/tryptophan ratio			R^2=0.06			R^2=0.46
<30 minutes	0.07	(-0.09, 0.22)	0.405	0.03	(-0.09, 0.15)	0.596
30-59 minutes	0.21	(0.08, 0.33)	0.001	0.11	(0.01, 0.21)	0.041
60-89 minutes	0.11	(-0.02, 0.25)	0.105	0.00	(-0.11, 0.11)	0.978
>90 minutes	0.07	(-0.08, 0.22)	0.345	0.01	(-0.10, 0.13)	0.823
Kynurenic acid			R^2=0.01			R^2=0.49
<30 minutes	-0.01	(-0.17, 0.15)	0.907	-0.02	(-0.14, 0.10)	0.724

30-59 minutes	-0.00	(-0.13, 0.12)	0.945	-0.13	(-0.22, -0.03)	0.012
60-89 minutes	0.12	(-0.03, 0.26)	0.112	0.02	(-0.09, 0.13)	0.744
>90 minutes	-0.03	(-0.18, 0.12)	0.658	-0.07	(-0.19, 0.04)	0.209

[1]hsCRP=high sensitive C-reactive protein. IL6=interleukin-6. IL10=interleukin-10. PAr-index= 4-pyridoxic acid divided/(pyridoxal 5´-phosphate + pyridoxal).
[2]Model: biomarker is the dependent variable. Sedentary time, age, sex, waist circumference, and creatinine are independent variables.
[3]For IL-6 and IL-10, Tobit regressions were used for the significance test and regular linear regressions to calculate the beta coefficients.
